# Selective case finding versus universal screening for detecting hypothyroidism in the first trimester of pregnancy: a comparative evaluation of a group of pregnant women from Rio de Janeiro

**DOI:** 10.20945/2359-3997000000209

**Published:** 2020-03-18

**Authors:** Tatiana Martins Benvenuto Louro Berbara, Nathalie Silva de Morais, Débora Ayres Saraiva, Carolina Martins Corcino, Annie Schtscherbyna, Karina Lúcia Moreira, Patrícia de Fátima dos Santos Teixeira, Mario Vaisman

**Affiliations:** 1 Faculdade de Medicina Hospital Universitário Clementino Fraga Filho Universidade Federal do Rio de Janeiro Rio de Janeiro RJ Brasil Departamento de Medicina Interna, Unidade de Endocrinologia, Faculdade de Medicina, Hospital Universitário Clementino Fraga Filho, Universidade Federal do Rio de Janeiro (HUCFF/UFRJ), Rio de Janeiro, RJ, Brasil

**Keywords:** Hypothyroidism, pregnancy, universal screening, case-finding

## Abstract

**Objective:**

Maternal hypothyroidism during pregnancy may lead to adverse outcomes. Recently published guidelines by the American Thyroid Association (ATA) do not advocate for universal screening but recommend a case-finding approach in high-risk pregnant women. The present study aims to evaluate the accuracy of this approach in identifying women with thyroid dysfunction during early pregnancy.

**Subjects and methods:**

This is a multiple-center, cross-sectional study. Three hundred and one pregnant women were enrolled. Anamnesis and a physical examination were performed to detect which women fulfilled the criteria to undergo laboratory screening of thyroid dysfunction, according to the ATA’s 2017 guidelines. The Zulewski’s validated clinical score was applied to assess signs and symptoms of hypothyroidism. Serum levels of thyrotropin (TSH), free thyroxine (FT4), anti-thyroperoxidase (TPO-Ab), and anti-thyroglobulin (Tg-Ab) antibodies were determined.

**Results:**

Two hundred and thirty one women (78%) were classified as high risk, and 65 (22%) were classified as low risk for thyroid dysfunction. Regarding the clinical score, 75 patients (31.2%) presented mild symptoms that were compatible with SCH, of which 22 (7.4%) had symptoms as the only risk factor for thyroid disease. 17 patients (5.7%) had SCH, of which 10 (58.8%) belonged to the high-risk group, and 7 (41.2%) belonged to the low-risk group. OH was found in 4 patients (1.4%): 3 (75%) in the high-risk group and 1 (25%) in the low-risk group.

**Conclusions:**

The ATA’s proposed screening criteria were not accurate in the diagnosis of thyroid dysfunction in pregnancy. Testing only the high-risk pregnant women would miss approximately 40% of all hypothyroid patients.

## INTRODUCTION

Maternal hypothyroidism, especially when diagnosed during the first trimester of gestation, may lead to adverse obstetric and neonatal outcomes as well as impaired neurocognitive development of the fetus (1-4). Recently published guidelines by the American Thyroid Association (ATA) do not advocate for universal screening but recommend case finding in high-risk pregnant women (5). According to this recommendation, all patients should undergo clinical evaluation during the first prenatal visit in order to identify the presence of risk factors for thyroid disease. If any of the risk factors, which were reviewed in the recent guidelines, are identified, testing for serum TSH during the first trimester of gestation is recommended (5).

Previously, the European guidelines did not advocate for universal screening because of the lack of grade 1 evidence, but they noted that the majority of the authors recommended it, considering the beneficial effects of levothyroxine (LT4) treatment on unknown overt hypothyroidism (OH) and the fact that the targeted approach will miss a large percentage of women with subclinical hypothyroidism (SCH) (6). Indeed, several studies have reported that case-based screening may result in failure to detect a large number of low-risk pregnant women with thyroid disease (7-9).

Besides the evidence that the case-finding approach may not be effective, universal thyroid screening during early pregnancy fulfils most criteria for a beneficial and cost-effective screening programme, according to recommendations of the WHO (10). Thyroid disease is an important health problem in pregnancy, which can be easily diagnosed with non-invasive and inexpensive diagnostic tests, and has a safe and well-tolerated treatment available. Screening all pregnant women has shown to be cost effective, even if only OH was considered to be associated with adverse obstetric outcomes (11).

The normal reference range for serum TSH concentrations in pregnancy, as well as the recommendations for LT4 treatment were also updated in the recent ATA guidelines. They reinforced the need to establish population-based trimester-specific reference ranges for serum TSH. When not available, the upper reference limit during the first trimester of pregnancy can be defined by reducing 0.5 mIU/L from the upper limit of TSH for non-pregnant women (5).

In Brazil, controversy regarding the best screening strategy of thyroid disease in pregnant women also exists. Therefore, we designed the present study to evaluate the accuracy of the ATA’s high-risk case-finding approach in identifying women with thyroid dysfunction during early pregnancy.

## SUBJECTS AND METHODS

This multiple-center, cross-sectional study was performed with an ongoing prospective cohort that included pregnant women attending prenatal programs in four public health care units from an urban area with iodine sufficiency (12) in a Brazilian coastal state. During the recruitment period, each unit was visited regularly, but at different times – not necessarily concomitantly. The average time of visit to each center was 10 to 12 months. All pregnant women who attended the units during our visit and met the study inclusion criteria were enrolled.

A total of 301 pregnant women attending at their first prenatal visit were included from September 2014 to January 2018. The recruiting criteria included age ≥ 18 years old, having a spontaneous pregnancy, and gestational age up to 12 weeks (defined by last menstrual period or ultrasound). Exclusion criteria were multifetal pregnancy, known autoimmune thyroid disease, or current use of levothyroxine, antithyroid drug, or nutritional supplements containing iodine. All subjects provided informed written consent, and the study was approved by the local research ethics committee (CAAE**:** 22546213.0.0000.5275).

From the initial sample of 301 pregnant women, 3 were excluded because they had a gestational age greater than 12 weeks, 1 was excluded because she was in treatment for hypothyroidism, and 1 was excluded because of a twin pregnancy. The final sample included 296 pregnant women.

Anamnesis and a general physical examination were performed to detect which pregnant women fulfilled the criteria for laboratory screening of thyroid dysfunction, according to 2017 ATA guidelines (5). The screening criteria are shown in table 1. If any of the criteria were identified, the patient was considered to be at high risk for thyroid dysfunction. Patients who did not meet any criteria were classified as low risk for thyroid dysfunction. The Zulewski’s validated clinical score was applied to assess signs and symptoms of hypothyroidism (13). According to this scoring system, women were considered clinically hypothyroid when they achieved more than 5 points; euthyroid with 0-2 points; and subclinically hypothyroid with 3-5 points (13). Body mass index (BMI) was calculated as weight (kg) divided by height squared (m^2^). As previously reported, the population studied was considered to have iodine sufficiency (12).

**Table 1:** Risk factors for thyroid dysfunction during pregnancy, according to ATA’sa 2017 guidelines

1. A history of hypothyroidism/hyperthyroidism or current symptoms/signs of thyroid dysfunction
2. Known thyroid antibody positivity or presence of a goiter
3. History of head or neck radiation or prior thyroid surgery
4. Age >30 years
5. Type 1 diabetes or other autoimmune disorders
6. History of pregnancy loss, preterm delivery, or infertility
7. Multiple prior pregnancies (≥ 2)
8. Family history of autoimmune thyroid disease or thyroid dysfunction
9. Morbid obesity (BMI ≥ 40 kg/m^2^)
10. Use of amiodarone or lithium, or recent administration of iodinated radiologic contrast
11. Residing in an area of known moderate to severe iodine insufficiency

^a^ American Thyroid Association.

Morning blood samples were collected from all patients to determine serum levels of thyrotropin (TSH), free thyroxine (FT4), anti-thyroperoxidase (TPO-Ab), and anti-thyroglobulin (Tg-Ab) antibodies. Diagnosis of OH required a serum TSH > 10.0 mIU/L or FT4 below the inferior reference range (associated with serum TSH elevations). Isolated hypothyroxinemia was defined by FT4 below the inferior range with normal TSH. TSH elevations above 3.8 mIU/L (and < 10.0 mIU/L) with normal FT4 constituted a diagnosis of SCH. We considered 3.8 mIU/L as the upper reference limit, by reducing 0.5 from the upper limit of TSH for non-pregnant women, according to recent recommendations from ATA’s guidelines (5). This value is similar to that found by Silva de Morais and cols.., who evaluated the TSH reference range in a group of pregnant women from Rio de Janeiro (14).

Serum TSH, FT4, TPO-Ab, and Tg-Ab were determined by an electrochemiluminescence immunometric assay with a Roche Modular Analytics^®^ E170 (Roche Diagnostics). The laboratory reference ranges of TSH were 0.4 to 4.3 mIU/L (for nonpregnant women), FT4 0.7 to 1.9 ng/dL, TPO-Ab < 34 IU/mL, and Tg-Ab < 115 IU/mL. The intra- and interassay coefficients of variation of serum TSH, FT4, TPO-Ab, and Tg-Ab were, respectively, 7.2% and 3%, 2.8% and 2.9%, 6.3% and 7.0 %, and 4.9% and 6.3%.

The associations between the risk classification and the occurrence of thyroid dysfunction and autoimmunity were evaluated by the Chi-square test. The observed p-values were obtained by Monte Carlo simulations (15) and corrected by the Sidàk procedure to multiple tests (16). In addition to the association analysis, we evaluated the sensitivity, specificity, positive and negative predictive values, and the accuracy of the ATA’s criteria for laboratory screening of thyroid dysfunction (5).

## RESULTS

We evaluated thyroid function and thyroid autoimmunity in 296 pregnant women. The characteristics of these women are shown in table 2.

**Table 2:** Baseline characteristics of the 296 pregnant women enrolled in the study:

Mean (± SD) maternal age (years)	28.8 (± 5.9)
Mean (± SD) gestational age at screening (weeks)	9.1 (± 2)
≥ 2 prior pregnancies, n (%)	76 (25.7)
History of previous miscarriages, n (%)	55 (19.1)
History of fetal death and/or preterm delivery, n (%)	10 (3.4)
History of infertility, n (%)	32 (11.9)
Family history of thyroid disease, n (%)	43 (14.3)
Personal history of other autoimmune diseases, n (%)	2 (0.7)
History of head/neck irradiation and/or thyroid surgery, n (%)	1 (0.4)
BMI^a^ ≥ 40, n (%)	3 (1)
Clinical euthyroidism (0 – 2 points in the Zulewski’s clinical score), n (%)	165 (68.8)
Signs and symptoms of SCH^b^ (3 – 5 points in the Zulewski’s clinical score), n (%)	75 (31.2)
Signs and symptoms of OH^c^ (> 5 points in the Zulewski’s clinical score), n (%)	0 (0)

^a^ body mass index; ^b^ subclinical hypothyroidism; ^C^ overt hypothyroidism.

Regarding the clinical score, 75 patients (31.2%) presented mild symptoms that were compatible with SCH, of which 22 (7.4%) had symptoms as the only risk factor for thyroid disease. Since no women had symptoms consistent with OH, we considered the presence of mild symptoms as a positive criteria for screening for thyroid dysfunction.

According to the criteria established by the 2017 ATA guidelines (5), 231 women (78%) were classified as high risk, and 65 (22%) were classified as low risk for thyroid dysfunction during pregnancy. In previous recommendations, ≥ 2 prior pregnancies were not considered one of the defining criteria of the high-risk group (17). According to the most recent guidelines, 10 women (3.4%) were newly classified as high risk for thyroid dysfunction, based on multiparity.

**Figure 1:**
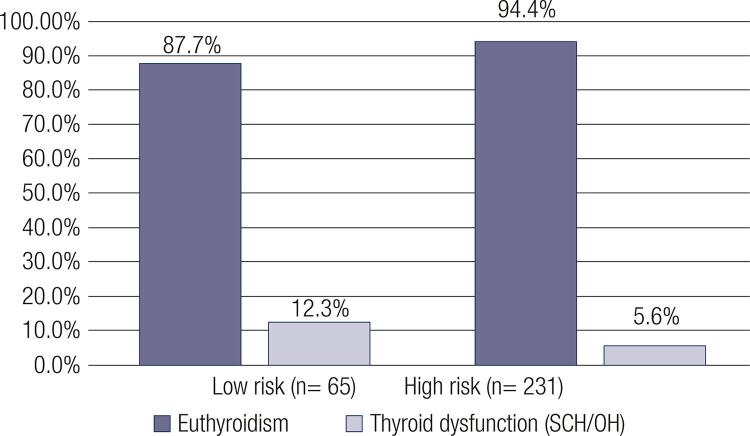
Prevalence of thyroid dysfunction according to risk classification

Regarding thyroid function, the median TSH was 1.42 mIU/L. SCH was found in 17 patients (5.7%), and OH was found in 4 patients (1.4%). The prevalence of thyroid dysfunction according to risk classification is shown in figure 1.

A total of 17 women (5.7%) were positive for TPO-Ab, and 17 of 276 (6.2%) were positive for Tg-Ab. 7 patients (2.54%) were positive for both TPO-Ab and Tg-Ab. 20 (6.8%) had no record of Tg-Ab values.

The associations between risk classification and occurrence of thyroid dysfunction, as well as the sensitivity, specificity, positive and negative predictive values, and accuracy of the ATA’s risk criteria are shown in table 3.

**Table 3:** Frequency distribution of individuals with thyroid dysfunction according to the risk classification, and the sensitivity, specificity, PPVa, NPVb, accuracy, and p-value

	High risk	Low risk	p-value	Sensitivity	Specificity	PPV^a^	NPV^b^	Accuracy
SCH^c^	10 (58.8%)	7 (41.2%)	0.232	58.8	20.8	4.3	89.2	23
OH^d^	3 (75%)	1 (25%)	0.999	75	21.9	1.3	98.5	22.6

^a^ positive predictive value; ^b^ negative predictive value; ^c^ subclinical hypothyroidism; ^d^ overt hypothyroidism.

Of 17 patients with SCH, 10 (58.8%) belonged to the high-risk group, and 7 (41.2%) belonged to the low-risk group. Sensitivity, specificity, positive predictive value (PPV), negative predictive value (NPV), and accuracy of the screening criteria were 58.8%, 20.8%, 4.3%, 89.2%, and 23%, respectively.

The criteria were also not sensitive (75%) or specific (21.9%) for the diagnosis of OH.

Additionally, the associations between risk classification and the presence of autoimmunity are shown in table 4.

**Table 4:** Frequency distribution of individuals with autoantibodies according to the risk classification, and the sensitivity, specificity, PPVa, NPVb, accuracy, and p-value

	High risk	Low risk	p-value	Sensitivity	Specificity	PPV^a^	NPV^b^	Accuracy
TPO-Ab^c^ positive	13 (76.5%)	4 (23.5%)	0.997	76.5	21.9	5.6	93.9	25
Tg-Ab^b^ positive	14 (82.4%)	3 (17.6%)	0.999	82.4	22.4	6.5	95.1	26.1

^a^ positive predictive value; ^b^ negative predictive value; ^c^ anti-thyroperoxidase; ^d^ anti-thyroglobulin.

No statistically significant association was observed between risk classification and thyroid dysfunction or presence of autoimmunity. A post-hoc analysis showed that the sample had a statistical power of 82% to detect small effect sizes (0.2) and 99.9% for medium effect sizes (0.5), according to Cohen’s criteria (18).

In its 2017 guidelines (5), the ATA suggests, based on moderate quality of evidence, that treatment with levothyroxine should be considered for pregnant women with TSH > 2.5 mIU/L, associated with the presence of autoantibodies. In our cohort, we found that 8 patients (2.7%) would receive thyroid hormone replacement according to this criteria: 4 patients in the high-risk group and 4 in the low-risk group.

## DISCUSSION

This multiple-center, cross-sectional study shows that the criteria proposed by the ATA’s 2017 guidelines do not represent a good screening tool for hypothyroidism in pregnancy. This was demonstrated by the absence of a significant association between risk classification and occurrence of SCH. In addition, the sensitivity found was below 60% and the specificity was around 20%. More important than these results are those of predictive values. The positive predictive value (PPV) was below 5%. Thus, when determining that a pregnant woman is at high risk, the probability of SCH is less than 5%. This result is of very limited utility in clinical practice. The negative predictive value (NPV), in turn, increased to around 90%, indicating that pregnant women in the low-risk group still presented a 10% probability of developing SCH.

Finally, we observed a low accuracy, showing a weak predictive capacity of the screening criteria in the diagnosis of SCH.

Regarding OH, there was also no significant association between risk and occurrence of overt thyroid dysfunction. The sensitivity, specificity, and PPV were 75%, 21.92%, and 1.3%, respectively. We found a high NPV (98.5%), but this result should be interpreted with caution, since it is probably due to the very low number of OH cases.

If the targeted high-risk case-finding approach is used, 7 women (41.2%) with SCH, and 1 woman (25%) with OH would be missed, since they belonged to the low-risk group. 4 women (50%) with positive autoantibodies and TSH > 2.5 mIU/L, for whom treatment with LT4 would be considered according to the new recommendations (5), would also be missed.

Rosario and cols. who evaluated 412 low-risk pregnant women in a metropolitan region of Belo Horizonte (Minas Gerais, Brazil) found that selective screening, recommended by ATA, does not result in a significant loss of women with an indication for LT4 treatment (19). These results differs from ours, possibly due to differences in patient selection, since it is unclear how assessment of signs and symptoms of hypothyroidism was made. In our study, 75 women (31.2%) had mild symptoms, according to Zulewski’s clinical score, of which 22 (7.4%) had symptoms as the only risk factor for thyroid disease. Considering that Brazil is a country of continental proportions, it is also possible that the divergences found are due to demographic differences, such as different profiles of iodine consumption. Our population was considered to have iodine sufficiency (12).

On the other hand, our results are similar to a number of other studies. Vaidya and cols., evaluated a cohort of 1560 pregnant women (7). Of all women with elevated TSH, 30% belonged to the low-risk group. In a retrospective cohort study performed in 2011 with data from the United States, Chang and cols. found that among 983 pregnant women in Boston, Massachusetts, up to 80% of women with elevated TSH levels might have been missed using a case-finding approach rather than universal screening (8). In 2015, Jouyandeh and cols. performed a systematic review and meta-analysis to compare the efficacy of universal screening versus a high-risk case-finding approach (9). Ten eligible articles were selected, and the results showed that the overall loss ratio in case-finding method was 49 % (CI 0.23–0.74).

Another important issue to be considered is that the screening strategy recommended by the ATA is poorly selective, since almost 80% of our sample would be screened for thyroid dysfunction, according to the targeted high-risk case-finding approach. In Brazil, the occurrence of multiparity is quite frequent, especially among women of lower socio-economic levels. This is demonstrated in the present study, since 76 women (25.7%) had previous history of ≥ 2 prior pregnancies. If we disconsider multiparity as a positive criteria for thyroid screening, 221 women (74.7%) would be classified as high risk, and 75 (25.3%) would be classified as low risk for thyroid dysfunction during pregnancy. Thus, the inclusion of this new criteria in the ATA’s most recent guidelines had little impact on the risk classification.

In our cohort, the prevalence of OH was 1.3% (n = 4), which is slightly higher than that found in the literature (20). Regarding SCH, 17 patients (5.7%) had TSH > 3.8mIU/L. The prevalence of subclinical thyroid disorder can vary widely, ranging from 2 to 35% of pregnancies, depending on the TSH reference values used to define SCH as well as by particularities of the population studied, such as iodine sufficiency and other demographic characteristics (20-22).

To our knowledge, this is the first study to evaluate the accuracy of the screening criteria proposed by the ATA in its most recent guidelines in the diagnosis of thyroid dysfunction, applying objective measurements of signs and symptoms of hypothyroidism, and in a population with known iodine sufficiency. All participants were included in their first trimester of gestation and had a comprehensive thyroid assessment including history, physical examination, and thyroid function tests.

For the first time, a validated clinical score was applied to assess signs and symptoms of thyroid dysfunction, which is one of the ATA’s criteria for case finding. Although the Zulewski’s clinical score has been validated for a non-pregnant population, Nazarpour and cols. showed in a recent study – published after the conclusion of our analyzes – that the Billewikz scoring system, which is an older version of the Zulewski’s score, may be a reliable tool in the diagnosis of OH during pregnancy (23). It is important to highlight 2 limitations of this study. First, it is the relatively small sample size. Second, our cohort may not be representative of other populations with differences in ethnicity, iodine intake, and TSH reference values.

In conclusion, the screening criteria proposed by the ATA’s 2017 guidelines were not accurate in the diagnosis of thyroid dysfunction in our cohort of pregnant women, since no significant association was found between risk classification and occurrence of SCH. Testing only the high-risk pregnant women in the targeted case-finding approach would miss around 40% of all hypothyroid patients.
